# Use of linezolid susceptibility test results as a surrogate for the susceptibility of Gram-positive pathogens to tedizolid, a novel oxazolidinone

**DOI:** 10.1186/s12941-014-0046-0

**Published:** 2014-09-20

**Authors:** Gary Zurenko, Paul Bien, Mekki Bensaci, Hina Patel, Grace Thorne

**Affiliations:** ZML Consulting, LLC, 7886 West C Avenue, Kalamazoo, MI 49009 USA; Cubist Pharmaceuticals, 4747 Executive Dr, San Diego, CA 92121 USA; Cubist Pharmaceuticals, 65 Hayden Ave, Lexington, MA 02421 USA

**Keywords:** Tedizolid, Linezolid, Oxazolidinone, *Staphylococcus aureus*, Antimicrobial susceptibility testing, Surrogate testing, Class representative

## Abstract

**Background:**

Tedizolid is a novel oxazolidinone antibacterial with potent activity against a wide range of Gram-positive pathogens, including methicillin-resistant *Staphylococcus aureus* and vancomycin-resistant enterococci. Although tedizolid is approved by the US Food and Drug Administration (FDA) for treatment of patients with acute bacterial skin and skin structure infection, commercial susceptibility testing products for tedizolid are not currently available. This study evaluated the usefulness of applying linezolid susceptibility test results as a surrogate for predicting susceptibility to tedizolid in clinically significant Gram-positive pathogens.

**Methods:**

Gram-positive isolates (N = 10,702) were obtained from annual surveillance programs conducted between 2009 and 2012, from 3 tedizolid clinical trials, and from a preclinical study of the antibacterial activity of tedizolid. Susceptibility testing of linezolid and tedizolid was performed using the reference broth microdilution method in accordance with Clinical and Laboratory Standards Institute methods.

**Results:**

The minimum inhibitory concentration (MIC) distribution for tedizolid and linezolid against this set of isolates was consistent with that of previous reports. Scatter plot analysis of relevant subsets of organisms was performed and showed high categorical agreement between linezolid and tedizolid MIC results (>99% for staphylococci and streptococci; >98% for enterococci). Very major error rates (ie, tedizolid false-susceptible errors) were very low and within acceptable limits for a surrogate agent: *S. aureus* and other staphylococcal species, 0%; *Enterococcus* spp, 0.2%; and *Streptococcus* spp, 0%.

**Conclusions:**

High categorical agreement between MIC values for tedizolid and linezolid and low very major error rates were shown for all organism groups tested, supporting the use of linezolid as a reliable surrogate for tedizolid susceptibility testing.

## Background

Tedizolid phosphate is a novel oxazolidinone prodrug antibacterial that is rapidly converted by endogenous phosphatases to the active moiety tedizolid. Tedizolid has potent activity against a wide range of Gram-positive pathogens, including methicillin-resistant *Staphylococcus aureus* (MRSA) and vancomycin-resistant enterococci (VRE) [1–4]. The efficacy of tedizolid phosphate 200 mg once daily for 6 days was shown to be noninferior to linezolid 600 mg twice daily for 10 days in each of 2 phase 3 clinical trials in patients with acute bacterial skin and skin structure infection (ABSSSI) [5,6]. In June 2014, the US Food and Drug Administration (FDA) approved tedizolid phosphate for treatment of patients with ABSSSI caused by certain susceptible Gram-positive pathogens [7].

For new antimicrobial agents, availability of commercial susceptibility testing products always lags behind drug approval by regulatory authorities because manufacturers of susceptibility testing products cannot seek FDA clearance to market their products until after drug approval. To facilitate antimicrobial susceptibility testing by clinical laboratories for a recently approved antimicrobial agent, an established agent from the same or similar class is often used as a surrogate to predict the susceptibility of clinical isolates to the new agent. Examples include the use of ceftriaxone for cefpodoxime [8]; carbapenem and third- and fourth-generation cephalosporins for ceftaroline [9]; vancomycin or teicoplanin for dalbavancin [10]; levofloxacin for gatifloxacin, moxifloxacin, and gemifloxacin [11]; and fluconazole or voriconazole for posaconazole [12].

Linezolid, the only other commercially available oxazolidinone, was first approved in the United States in 2000 and is now widely available in multiple commercial susceptibility testing products. We evaluated the usefulness of linezolid as a surrogate agent for predicting the susceptibility of clinically significant Gram-positive pathogens to tedizolid.

## Material and methods

### Sources for bacterial strains

A total of 10,702 Gram-positive isolates were obtained from annual surveillance programs, from 3 clinical trials of tedizolid for treatment of ABSSSI, and from a preclinical study of tedizolid activity that included linezolid-resistant isolates. The surveillance isolates included 9022 Gram-positive clinical isolates collected from sites in the United States, Belgium, Czech Republic, France, Germany, Hungary, Italy, Spain, Sweden, and United Kingdom from 2009 through 2012. A total of 937 isolates were collected between 2008 and 2013 from a US-based phase 2 and from 2 multinational phase 3 clinical trials of tedizolid (NCT01519778, NCT0117022, and NCT01421511). An additional 743 isolates were collected in the United States as part of a preclinical study to evaluate the antibacterial activity of tedizolid [1]. Overall, the isolates included in this study were composed of the following groupings: *S. aureus* (n = 7187), staphylococci other than *S. aureus* (n = 674), *Enterococcus* spp (n = 1241), and *Streptococcus* spp (n = 1600).

### Susceptibility testing

Susceptibility testing was performed using the reference broth microdilution method in accordance with the guidelines of the Clinical and Laboratory Standards Institute (CLSI) [13]. Tedizolid was supplied by Cubist Pharmaceuticals (San Diego, CA); ThermoFisher Scientific (Cleveland, OH) produced the broth microdilution panels for the annual surveillance programs and the clinical trials; Clinical Microbiology Institute (Wilsonville, OR) produced the broth microdilution panels for the preclinical study [1]. Assay performance was monitored using CLSI-recommended quality control strains *S. aureus* ATCC 29213, *Enterococcus faecalis* ATCC 29212, and *Streptococcus pneumoniae* ATCC 49619.

### Analyses

For select organism groupings, the minimum inhibitory concentration (MIC) results for tedizolid were directly compared with those for linezolid using scatter plots. The FDA approved the following tedizolid breakpoints: ≤0.5 μg/ml (susceptible), 1 μg/ml (intermediate), and ≥2 μg/ml (resistant) for *S. aureus* (including MRSA isolates); ≤0.5 μg/ml (susceptible) for *Streptococcus pyogenes*, *Streptococcus agalactiae*, and *E. faecalis*; and ≤0.25 μg/ml (susceptible) for *Streptococcus anginosus* group isolates (ie, *S. anginosus, S. intermedius,* and *S. constellatus*) [7]. For the analyses presented in the current study, the following tedizolid breakpoints were applied: ≤0.5 μg/ml (susceptible), 1 μg/ml (intermediate), and ≥2 μg/ml (resistant) for staphylococci; ≤0.5 μg/ml (susceptible) and ≥1 μg/ml (nonsusceptible) for streptococci and enterococci; and ≤0.25 μg/ml (susceptible) and ≥0.5 μg/ml (nonsusceptible) for analysis of only the *S. anginosus* group isolates. Current CLSI breakpoints were applied for linezolid [14].

The definitions of errors in these analyses were (1) very major error (ie, a false-susceptible error in which a susceptible result was obtained for linezolid and a resistant [or nonsusceptible] result was obtained for tedizolid); (2) major error (ie, a false-resistant error in which a resistant [or nonsusceptible] result was obtained for linezolid and a susceptible result was obtained for tedizolid); and (3) minor error (ie, result for one of the agents was intermediate and the other agent was susceptible, nonsusceptible, or resistant) [15]. Error rates were calculated separately for each organism grouping using the total number of isolates in the respective grouping as the denominator.

## Results

### MIC distributions

Table 1 summarizes the cumulative inhibition across the MIC range stratified by organism grouping for tedizolid and linezolid. MIC results for the CLSI quality control strains were within CLSI-recommended ranges for both test agents (data not shown) [14]. The minimum inhibitory concentrations required to inhibit the growth of 90% of organisms (MIC_90_ values) were 0.5 and 2 μg/ml or less for tedizolid and linezolid, respectively. Isolates resistant to linezolid (*S. aureus* [n = 20], non-*S. aureus* staphylococci [n = 6], *Enterococcus* spp [n = 4], and *Streptococcus* spp [n = 1]) were predominantly contributed by the preclinical study [1]. Linezolid-resistant isolates were rarely isolated in surveillance studies and were not present among patients in the clinical program.

**Table 1:** **Cumulative inhibition at MIC values by organism grouping and antimicrobial agent**

**Organism grouping (no. tested)**	**Cumulative number (%) of isolates inhibited at MIC value (μ** **g/ml)**								
**Antimicrobial agent**	**≤0.06**	**0.12**	**0.25** ^**a**^	**0.5**	**1**	**2**	**4**	**8**	**>8**
*S. aureus* (7187)									
Tedizolid	17 (0.2)	550 (7.7)	*5024(69.9)*	**7163 (99.6)**	7175 (99.8)	7180 (99.9)	7184 (99.9)	7186 (99.9)	7187 (100)
Linezolid	--	--	7 (0.1)	29 (0.4)	1580 (21.9)	***6813 (94.7)***	7167 (99.7)	7170 (99.7)	7187 (100)
Staphylococcal species (non-*S. aureus*)^b^ (674)									
Tedizolid	30 (4.5)	323 (47.9)	*597 (88.5)*	**664 (98.5)**	669 (99.2)	669 (99.2)	673 (99.8)	674 (100)	674 (100)
Linezolid	--	--	6 (0.9)	163 (24.1)	*575 (85.3)*	**660 (97.9)**	668 (99.1)	669 (99.2)	674 (100)
*Enterococcus* spp. (1241)									
Tedizolid	8 (0.6)	71 (5.7)	*701 (56.4)*	**1224 (98.6)**	1237 (99.6)	1240 (99.9)	1241 (100)	1241 (100)	1241 (100)
Linezolid	--	--	5 (0.4)	39 (3.1)	544 (43.8)	***1219 (98.2)***	1237 (99.6)	1238 (99.7)	1241 (100)
*Streptococcus* spp. (1600)									
Tedizolid	80 (5.0)	790 (49.3)	***1592 (99.5)***	1600 (100)	1600 (100)	1600 (100)	1600 (100)	1600 (100)	1600 (100)
Linezolid	--	--	56 (3.5)	474 (29.6)	***1516 (94.7)***	1599 (99.9)	1600 (100)	1600 (100)	1600 (100)
*Streptococcus anginosus* group^c^ (91)									
Tedizolid	35 (38.4)	*75 (82.4)*	**91 (100)**	91 (100)	91 (100)	91 (100)	91 (100)	91 (100)	91 (100)
Linezolid	--	--	35 (38.4)	*67 (73.6)*	**91 (100)**	91 (100)	91 (100)	91 (100)	91 (100)

*Abbreviations: MIC* minimum inhibitory concentration, *MIC*_*50*_ MIC required to inhibit growth of 50% of isolates, *MIC*_*90*_ MIC required to inhibit growth of 90% of isolates.

Italics indicate MIC_50_.

Bolding indicates MIC_90_.

^a^An MIC value of 0.25 was the lowest MIC value tested for linezolid. Linezolid results should be read as ≤0.25 μg/ml.

^b^Includes isolates of *S. capitis* (28), *S. caprae* (4), *S. cohnii* (2), *S. epidermidis* (405), *S. haemolyticus* (52), *S. hominis* (56), *S. intermedius* (7), *S. lugdunensis* (47), *S. pasteuri* (1), *S. pettenkoferi* (1), *S. saprophyticus* (28), *S. schleiferi* (6), *S. simulans* (14), *S. warneri* (12), *S. xylosus* (2), and unspeciated coagulase-negative *Staphylococcus* spp. (9).

^c^Includes isolates of *S. anginosus*, *S. intermedius*, and *S. constellatus*.

### Linezolid as a surrogate for tedizolid susceptibility when testing *S. aureus* isolates

**Figure 1:**
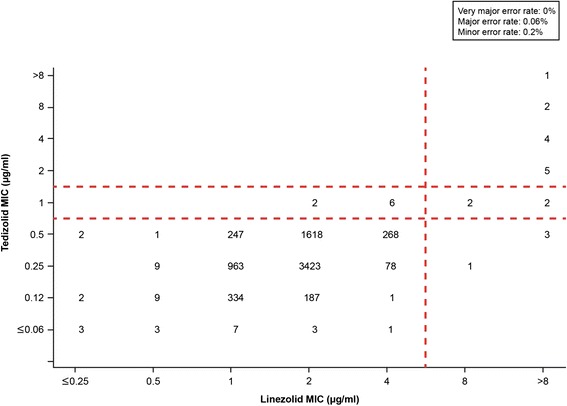
**Scatter plot comparing tedizolid and linezolid minimum inhibitory concentration (MIC) values for 7187** ***Staphylococcus aureus*** **isolates.** Dashed lines represent the breakpoints for tedizolid (≤0.5 μg/ml [susceptible], 1 μg/ml [intermediate], and ≥2 μg/ml [resistant]) and linezolid (≤4 μg/ml [susceptible] and ≥8 μg/ml [resistant]).

For 7187 *S. aureus* isolates*,* the very major error rate was 0%, with no isolate showing susceptibility to linezolid while being resistant to tedizolid (Figure 1). A major error (false-resistant error) was shown for 4 isolates (0.06%). A minor error was noted for 12 isolates (0.2%) that showed intermediate susceptibility to tedizolid but were either linezolid susceptible (n = 8) or linezolid resistant (n = 4). Categorical agreement was 99.8% (7171 of 7187 isolates).

### Linezolid as a surrogate for tedizolid susceptibility when testing staphylococcal isolates other than *S. aureus*

**Figure 2:**
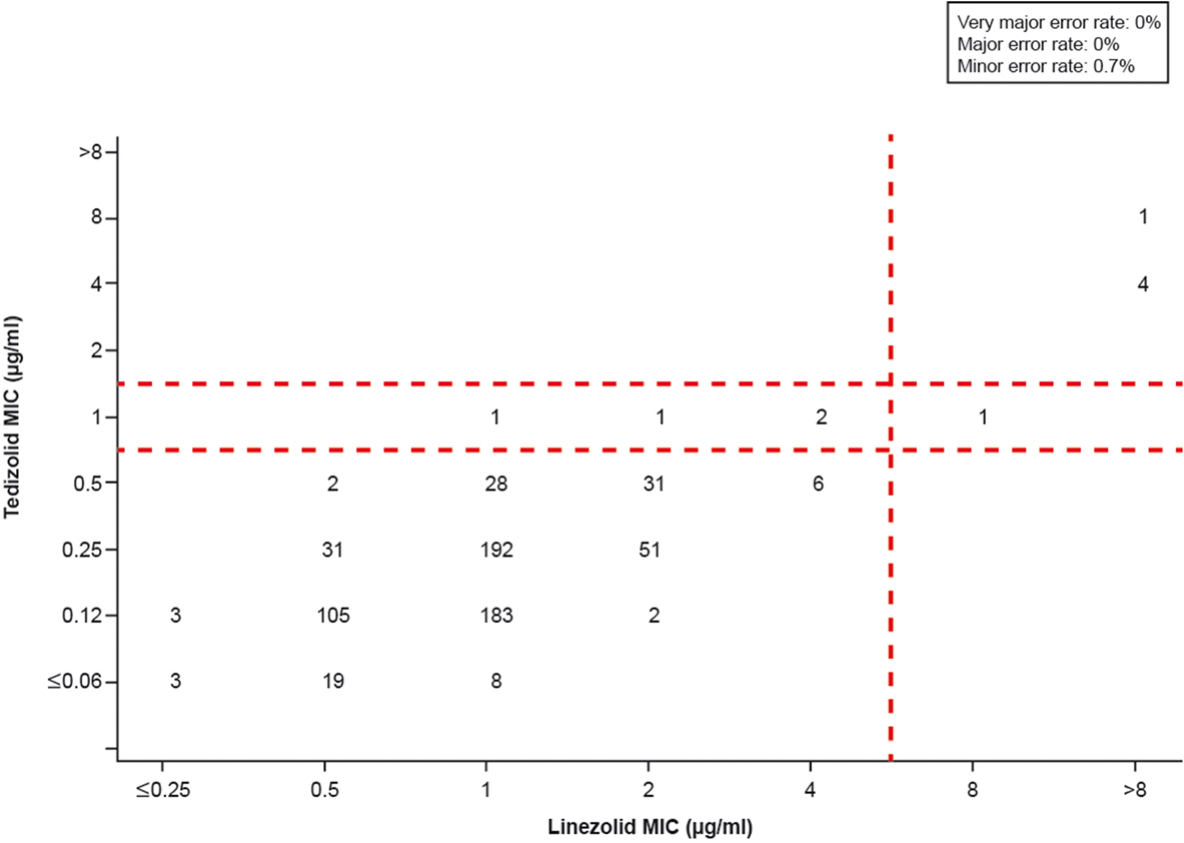
**Scatter plot comparing tedizolid and linezolid minimum inhibitory concentration (MIC) values for 674 staphylococcal isolates other than** ***Staphylococcus aureus.*** Dashed lines represent the US Food and Drug Administration–approved *S. aureus* breakpoints for tedizolid generalized for all staphylococci (≤0.5 μg/ml [susceptible], 1 μg/ml [intermediate], and ≥2 μg/ml [resistant]) and the Clinical and Laboratory Standards Institute–approved breakpoints for linezolid (≤4 μg/ml [susceptible] and ≥8 μg/ml [resistant]). These isolates are primarily from coagulase-negative staphylococcal species (eg, *S. epidermidis, S. haemolyticus*, *S. lugdunensis*), though some isolates are from coagulase-positive staphylococcal species other than *S. aureus*.

Among 674 isolates of staphylococcal species other than *S. aureus*, no very major errors (false-susceptible) or major errors (false-resistant) were noted when breakpoints ≤0.5 μg/ml (susceptible), 1 μg/ml (intermediate), and ≥2 μg/ml (resistant) were applied (Figure 2). A minor error was reported for 5 isolates (0.7%), which showed intermediate susceptibility to tedizolid but were susceptible to linezolid (n = 4) or resistant to linezolid (n = 1). Categorical agreement was 99.3% (669 of 674 isolates). The staphylococcal isolates in this organism grouping were predominantly from coagulase-negative species.

### Linezolid as a surrogate for tedizolid susceptibility when testing enterococcal isolates

**Figure 3:**
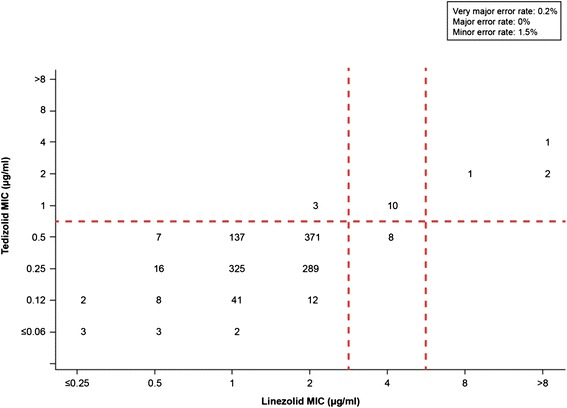
**Scatter plot comparing tedizolid and linezolid minimum inhibitory concentration (MIC) values for 1241 enterococcal isolates.** Dashed lines represent the US Food and Drug Administration–approved *Enterococcus faecalis* breakpoint for tedizolid generalized for all enterococci (≤0.5 μg/ml [susceptible]) and the Clinical and Laboratory Standards Institute–approved breakpoints for linezolid (≤2 μg/ml [susceptible], 4 μg/ml [intermediate], and ≥8 μg/ml [resistant]).

To analyze the 1241 enterococcal isolates (predominantly isolates of *E. faecalis* and *E. faecium,* including vancomycin-resistant isolates of both species), a breakpoint ≤0.5 μg/ml (susceptible) was applied (Figure 3). Three isolates (0.2%) exhibited a very major error, which were susceptible to linezolid (MIC, 2 μg/ml) but nonsusceptible to tedizolid (MIC, 1 μg/ml). A minor error was reported for 18 isolates (1.5%), which displayed intermediate resistance to linezolid but were susceptible (n = 8) or nonsusceptible (n = 10) to tedizolid. Categorical agreement was 98.3% (1220 of 1241 isolates).

### Linezolid as a surrogate candidate for streptococci

**Figure 4:**
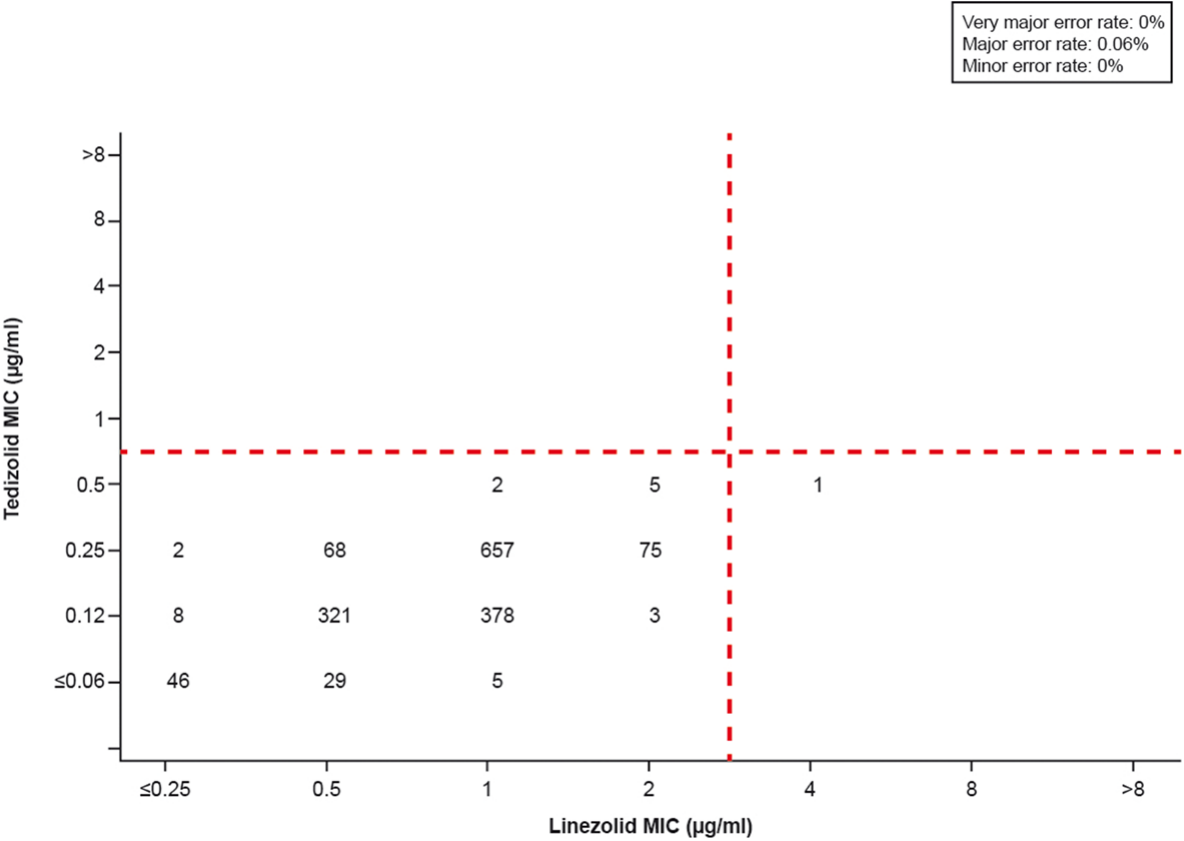
**Scatter plot comparing tedizolid and linezolid minimum inhibitory concentration (MIC) values for 1600 streptococcal isolates.** Dashed lines represent the US Food and Drug Administration–approved *Streptococcus pyogenes* and *Streptococcus agalactiae* breakpoint for tedizolid generalized for all streptococci (≤0.5 μg/ml [susceptible]) and the Clinical and Laboratory Standards Institute–approved breakpoint for linezolid (≤2 μg/ml [susceptible]).

All streptococcal isolates tested (n = 1600) were susceptible to tedizolid when a breakpoint ≤0.5 μg/ml (susceptible) was applied (Figure 4). The streptococcal isolates included a mix of species, including *S. pyogenes*, *S. agalactiae*; Lancefield groups C, F, and G; and viridans group isolates. A major error was seen for a single isolate of *S. agalactiae* (0.06%), which was linezolid resistant (MIC, 4 μg/ml) but tedizolid susceptible (MIC, 0.5 μg/ml). There were no very major errors or minor errors. Categorical agreement was 99.9% (1599 of 1600 isolates).

**Figure 5:**
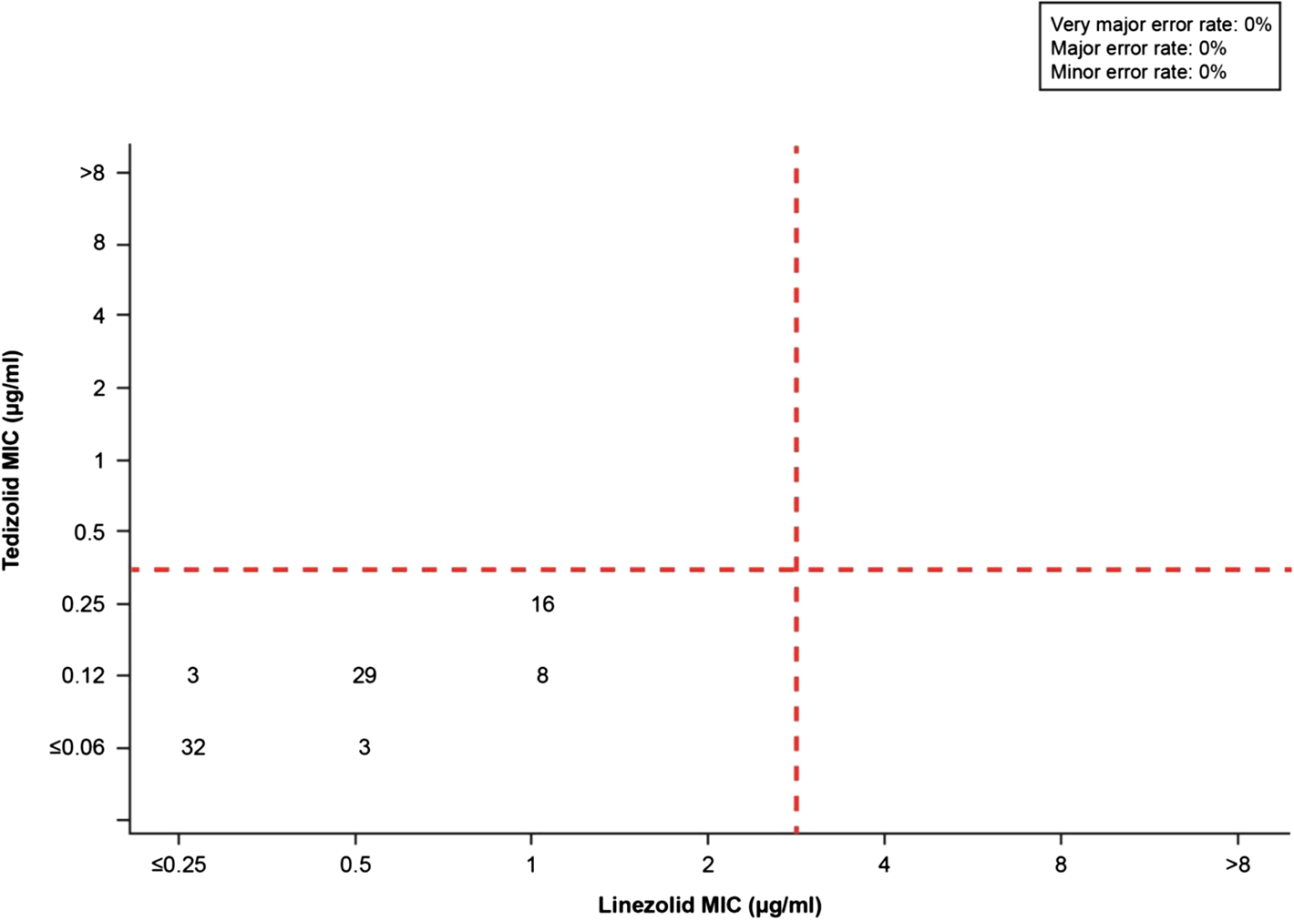
**Scatter plot comparing tedizolid and linezolid minimum inhibitory concentration (MIC) values for 91** ***Streptococcus anginosus*** **group isolates.** Dashed lines represent the US Food and Drug Administration–approved breakpoint for tedizolid for *S. anginosus* group isolates (≤0.25 μg/ml [susceptible]) and the Clinical and Laboratory Standards Institute–approved breakpoint for linezolid (≤2 μg/ml [susceptible]).

The subset of isolates (n = 91) confirmed to be members of the *S. anginosus* group (ie, *S. anginosus*, *S. constellatus*, and *S. intermedius*) were analyzed separately using the FDA-approved breakpoint for tedizolid of ≤0.25 μg/ml (susceptible) (Figure 5). All isolates were susceptible to tedizolid and linezolid both by these criteria; therefore, there were no interpretive errors.

## Discussion

Tedizolid is a novel oxazolidinone approved by the FDA for management of ABSSSI caused by certain susceptible Gram-positive pathogens, and is in further clinical development for management of nosocomial pneumonia. Tedizolid represents a potentially valuable therapeutic addition to the antimicrobial agents currently used for management of ABSSSI; however, FDA-approved commercial products for tedizolid susceptibility testing are currently unavailable. This analysis demonstrated that linezolid, the only oxazolidinone available in commercial susceptibility testing products [16], can serve as a surrogate agent to predict tedizolid susceptibility of clinically relevant Gram-positive pathogens.

For all organism groupings tested (*S. aureus,* staphylococci other than *S. aureus, Enterococcus* spp, *Streptococcus* spp, and *S. anginosus* group), linezolid susceptibility was highly predictive of tedizolid susceptibility. For all pathogen groups, the categorical agreement between test results for tedizolid and linezolid was >98%, and rates of very major and major errors were well within the CLSI limits of acceptability (ie, very major and major error rates of ≤1.5% and ≤3%, respectively) [15]. Recommendations from previous studies indicate that absolute categorical agreement rates of ≥90% are essential and rates of ≥95% are preferred [10,12]. In this study, the risk for very major error where an isolate would be reported as susceptible despite being resistant or nonsusceptible was rare and was highest for *Enterococcus* spp, which had a reported very major error rate of only 0.2%. The FDA-approved breakpoints for tedizolid were derived from MIC population distributions, pharmacokinetic/pharmacodynamics information, Monte Carlo simulations, and clinical and microbiological outcome data from the ABSSSI clinical program.

The main mechanisms for linezolid resistance consist of mutations in chromosomal genes encoding 23S ribosomal RNA and ribosomal proteins L3 and L4, or the presence of the plasmid-borne *cfr* gene. Although tedizolid MIC values are affected by chromosomal mutations, tedizolid has been shown to retain antimicrobial activity against strains with the *cfr* gene [17–19]. When linezolid is used as a surrogate testing agent for tedizolid, *cfr*-positive isolates will likely result in major errors (false resistance or false nonsusceptible). In these rare instances, the major errors should not adversely affect patient care because a laboratory result of resistant or nonsusceptible to tedizolid should preclude the use of the agent in these situations.

Clinical laboratories have successfully used a class representative to predict susceptibility to other agents in the same class as a practical alternative when specific susceptibility testing products are unavailable [8–12]. Given the lack of such products for tedizolid, this approach provides a useful strategy to assist in the appropriate therapeutic use of tedizolid against clinically important Gram-positive pathogens. As commercial tedizolid susceptibility products become available, they could rapidly replace the interim use of linezolid as the surrogate testing agent.

## Conclusions

This analysis showed that, for all Gram-positive pathogen groups tested, there was high categorical agreement between tedizolid and linezolid susceptibility results, and rates of very major errors and major errors were well within acceptable limits. These findings show that linezolid can be used as a reliable surrogate to predict susceptibility testing results for tedizolid against clinically relevant Gram-positive pathogens. Consideration should be given to the use of linezolid as a surrogate so susceptibility testing results for tedizolid can be made rapidly available to health care providers.
